# Estradiol and Progesterone as key modulators of central systems implicated in schizophrenia

**DOI:** 10.1192/j.eurpsy.2022.1897

**Published:** 2022-09-01

**Authors:** A. Ruiz-Tristan, C. Rivera-Clemente, V. Gomez-De-Las-Heras, S. Ávila, J. Molina

**Affiliations:** 1 Hospital 12 de Octubre, Villaverde Mental Health Center, Madrid, Spain; 2 Hospital 12 de Octubre, Psychiatry, Madrid, Spain; 3 Complutense University of Madrid, Psychiatry, Madrid, Spain; 4 Hospital 12 de Octubre, Center For Biological Research In Mental Health (cibersam), Madrid, Spain; 5 Hospital 12 de Octubre, Clinical Management Area Of Psychiatry And Mental Health, Madrid, Spain

**Keywords:** schizophrénia, progesterone, estradiol, endocrinology

## Abstract

**Introduction:**

It has been suggested that dysregulation of sex hormones is associated with schizophrenia. However, obesity and metabolic syndrome are very common between schizophrenic patients, and it can also dysregulate sex hormones so they could act as confounders.

**Objectives:**

To determine if estradiol and progesterone are abnormally elevated regardless of obesity or metabolic syndrome in men with SCZ.

**Methods:**

We measured serum levels of progesterone and estradiol in 56 schizophrenic male patients at treatment with a depot antipsychotic. Subsequently, we studied the association or independence of our results with obesity or metabolic syndrome by a Chi Square Test.

**Results:**

66.07% of our patients elevated progesterone levels, 19.64% of our patients elevated estradiol levels, and 16.07% of our patients elevated both, progesterone and estradiol, simultaneously. We found no relationship between increased estradiol and / or progesterone with obesity and / or metabolic syndrome.

**Conclusions:**

Estradiol and progesterone are abnormally elevated regardless of obesity and / or metabolic syndrome in male schizophrenic patients on depot treatment.

**Disclosure:**

No significant relationships.

